# CASNet: curvature-aware cardiac MRI segmentation with multi-scale and attention-driven encoding for enhanced risk-oriented structural analysis

**DOI:** 10.3389/fmed.2025.1688872

**Published:** 2026-01-21

**Authors:** Yan Du, Kaisen Huang, Miaomiao Yue, Cheng Chen, Xiaojian Deng, Ning Wang

**Affiliations:** 1 Department of Cardiology, Deyang People's Hospital, Deyang, Sichuan, China; 2 Department of Clinical Medicine, School of Clinical Medicine, Southwest Medical University, Luzhou, Sichuan, China; 3 School of Medical Information and Engineering, Southwest Medical University, Luzhou, Sichuan, China

**Keywords:** cardiac MRI segmentation, curvature-aware loss, deep learning, medical image analysis, multi-scale context

## Abstract

Accurate segmentation of cardiac structures in magnetic resonance imaging (MRI) is essential for reliable diagnosis and quantitative analysis of cardiovascular diseases. However, conventional convolutional neural networks often struggle to maintain both semantic consistency and geometric smoothness, particularly in challenging slices with high anatomical variability. In this work, we propose CASNet, a novel U-Net-based architecture that integrates three key enhancements to address these limitations. First, we introduce a Multi-Scale Context Block (MSCB) at the network bottleneck to enrich encoder features with diverse receptive fields, enabling robust representation of cardiac structures across varying spatial scales. Second, we replace standard skip connections with Cross-Attentive Skip Connections (CASC), allowing the decoder to selectively aggregate spatial features from encoder layers via attention-weighted fusion. This mitigates semantic mismatch and promotes more effective feature reuse. Third, we incorporate a Curvature-Aware Loss that penalizes second-order spatial discontinuities in the predicted segmentation, thereby improving the smoothness and anatomical plausibility of the boundaries. Extensive experiments on the ACDC dataset demonstrate that CASNet outperforms baseline U-Net models and recent attention-based architectures, achieving superior performance in both region overlap and boundary accuracy metrics. The proposed approach provides a robust and generalizable solution for high-precision cardiac MRI segmentation, which may serve as a foundation for future downstream clinical applications in AI-assisted cardiac analysis.

## 1 Introduction

Cardiovascular diseases (CVDs) remain the leading cause of morbidity and mortality worldwide (1–3). Accurate segmentation of cardiac structures in magnetic resonance imaging (MRI) is crucial for the assessment of cardiac function and disease progression (4, 5). Reliable delineation of regions such as the left ventricle (LV), right ventricle (RV), and myocardium (MYO) enables quantitative evaluation of ventricular volumes, ejection fraction, and myocardial mass, which are essential clinical indicators (6). However, automated cardiac segmentation remains a challenging task due to several factors: the large anatomical variability between patients, poor contrast at boundaries, the presence of pathological changes, and the variability across different slices from apex to base.

In recent years, deep convolutional neural networks (CNNs), particularly encoder—decoder architectures like U-Net, have shown remarkable success in medical image segmentation (7, 8). These networks extract hierarchical features through a series of downsampling and upsampling layers, enabling both low-level spatial detail preservation and high-level semantic abstraction. Nevertheless, traditional U-Net suffers from two critical limitations that hinder its segmentation performance in cardiac MRI (9, 10).

First, standard U-Net applies a fixed-size convolution kernel at each layer, limiting its receptive field and capacity to model multi-scale anatomical structures (11). This becomes problematic in cardiac MRI, where structural patterns vary significantly across slices. Various studies have attempted to address this issue by introducing pyramid pooling modules, atrous convolutions, or hybrid multi-branch networks (12, 13). While these approaches improve scale-awareness, they are often placed in the decoder or final prediction layers, leaving the encoder bottleneck underutilized in terms of contextual modeling.

Second, the skip connections in U-Net directly concatenate encoder and decoder features at corresponding resolutions without any feature selection or semantic alignment (14, 15). This naive fusion assumes that encoder features, which are often dominated by low-level edge and texture cues, are compatible with the semantically richer decoder features. In practice, this assumption frequently leads to semantic inconsistencies and noisy feature propagation, especially in regions with weak contrast or ambiguous boundaries (16). Attention mechanisms have been introduced to re-weight channel or spatial features, but many methods focus on self-attention within single feature maps rather than cross-level interaction (17, 18).

In addition to representation issues, most existing methods use pixel-wise loss functions such as Dice or binary cross-entropy (BCE), which optimize for region-level overlap but do not explicitly enforce geometric regularity. As a result, predicted boundaries can appear jagged or fragmented, particularly in small or thin structures such as the myocardium. This raises concerns about the anatomical plausibility and clinical utility of the segmentation outputs (19, 20).

To address these challenges, we propose a novel architecture named CASNet, which incorporates three key innovations. Motivated by the need for robust multi-scale representation, we introduce a multi-scale context block at the bottleneck layer of the encoder. This module captures fine-to-coarse features using parallel convolutions of varying kernel sizes and fuses them to enrich semantic information before decoding. To bridge the semantic gap between encoder and decoder features, we design cross-attentive skip connections, allowing the decoder to selectively query and aggregate spatially relevant encoder features via a learned attention mechanism. Finally, to enhance the geometric consistency of the predicted contours, we introduce a curvature-aware loss term that penalizes high-frequency changes in the segmentation boundary by regularizing second-order spatial derivatives.

These modules are carefully integrated into a U-Net backbone, forming a unified framework capable of learning discriminative and structure-preserving representations for cardiac MRI segmentation. Our method is evaluated on the public ACDC dataset and demonstrates superior performance over existing baselines in terms of both region-based and boundary-aware metrics.

The main contributions of this work are as follows:

- We propose a multi-scale context block (MSCB) placed at the bottleneck layer, which enriches the encoder output with multi-receptive field features to improve the robustness of semantic representation.
- We design cross-attentive skip connections (CASC) that replace naive feature concatenation with attention-guided feature selection, enhancing the semantic compatibility between encoder and decoder layers.
- We introduce a curvature-aware loss function that regularizes boundary smoothness by penalizing second-order curvature changes, leading to anatomically plausible segmentation with better boundary quality.

## 2 Related work

Deep learning has substantially advanced medical image segmentation, particularly through the use of convolutional neural networks (CNNs), which offer strong capabilities in hierarchical feature learning (21, 22). U-Net (23) and its numerous variants have become the backbone for a wide range of tasks, including cardiac MRI segmentation, due to their encoder–decoder symmetry and skip connections that balance semantic abstraction and spatial precision (24, 25). Nonetheless, challenges persist in capturing multi-scale anatomical structures, preserving boundary smoothness, and mitigating semantic gaps between encoder and decoder features.

One major research direction focuses on multi-scale context modeling. This is especially important in cardiac segmentation due to substantial inter-slice anatomical variation. To enhance scale sensitivity, UNet++ (26) employed dense nested pathways, while methods such as DeepLabV3 (27), CE-Net (28), and other studies (29) introduced atrous spatial pyramid pooling, pyramid pooling, or dilated convolutions. However, most of these designs apply multi-scale modules at the decoder or output stages, which may limit their impact on early semantic feature encoding. In contrast, the Multi-Scale Context Block (MSCB) in our framework is embedded in the encoder bottleneck, enriching deep features with varied receptive fields to improve upstream contextual representation.

Another research line involves the use of attention mechanisms to enhance feature selection and fusion. AG-UNet (30), SE-Net (31), and CBAM (32) introduced channel-wise or spatial attention in different parts of the encoder-decoder pipeline. More recent models have employed self-attention for long-range dependency modeling (33, 34). Most of these approaches focus on internal attention within feature maps. In contrast, our Cross-Attentive Skip Connections (CASC) introduce cross-attention between decoder queries and encoder keys/values, enabling the decoder to selectively retrieve spatially relevant encoder features. This approach is inspired by transformer designs and aligns with recent efforts to bridge encoder–decoder gaps via attention.

Boundary and shape-aware supervision is another critical direction. While pixel-wise losses such as Dice and binary cross-entropy remain standard, they do not penalize geometric irregularities in segmentation masks (35). Prior work has introduced contour-aware losses, distance transform penalties, and active contour formulations to enforce structural plausibility. Our proposed curvature-aware loss takes a different approach by regularizing second-order derivatives through the Hessian matrix. This penalizes local curvature inconsistency and promotes smooth, anatomically realistic boundaries.

Transformer-based models such as Swin-Unet (36) and MedT (37) have also demonstrated strong performance in medical segmentation tasks by modeling non-local dependencies. However, these models often require extensive training data and high computational cost. Recent efforts to integrate transformers into CNN backbones (38–40) have led to hybrid designs that aim to balance global modeling and efficiency. Notably, recent work has explored anatomical structure-aware transformers for cardiac MRI segmentation (41), demonstrating the value of incorporating prior anatomical knowledge into transformer architectures. Block-partitioned transformer designs with global-local information integration have also shown promise in cardiac segmentation tasks (42). Additionally, transformer-based models have been assessed for detecting cardiac systolic abnormalities in catheterization imaging (43), highlighting their growing adoption in diverse cardiac imaging modalities. While these transformer-based approaches are promising, they may introduce architectural complexity or computational overhead that limits clinical applicability. CASNet instead retains a CNN-compatible design, incorporating lightweight cross-attention mechanisms while maintaining practical feasibility and efficiency.

Finally, there is growing interest in the integration of segmentation with downstream diagnostic tasks. (44) combined deep features and radiomics to improve brain tumor grading, highlighting the relevance of segmentation quality to subsequent clinical decision-making. Although our work focuses on methodological contributions to segmentation, improvements in anatomical accuracy may benefit future downstream applications in AI-assisted cardiology and risk stratification.

## 3 Methodology

### 3.1 Model overview

**Figure 1:**
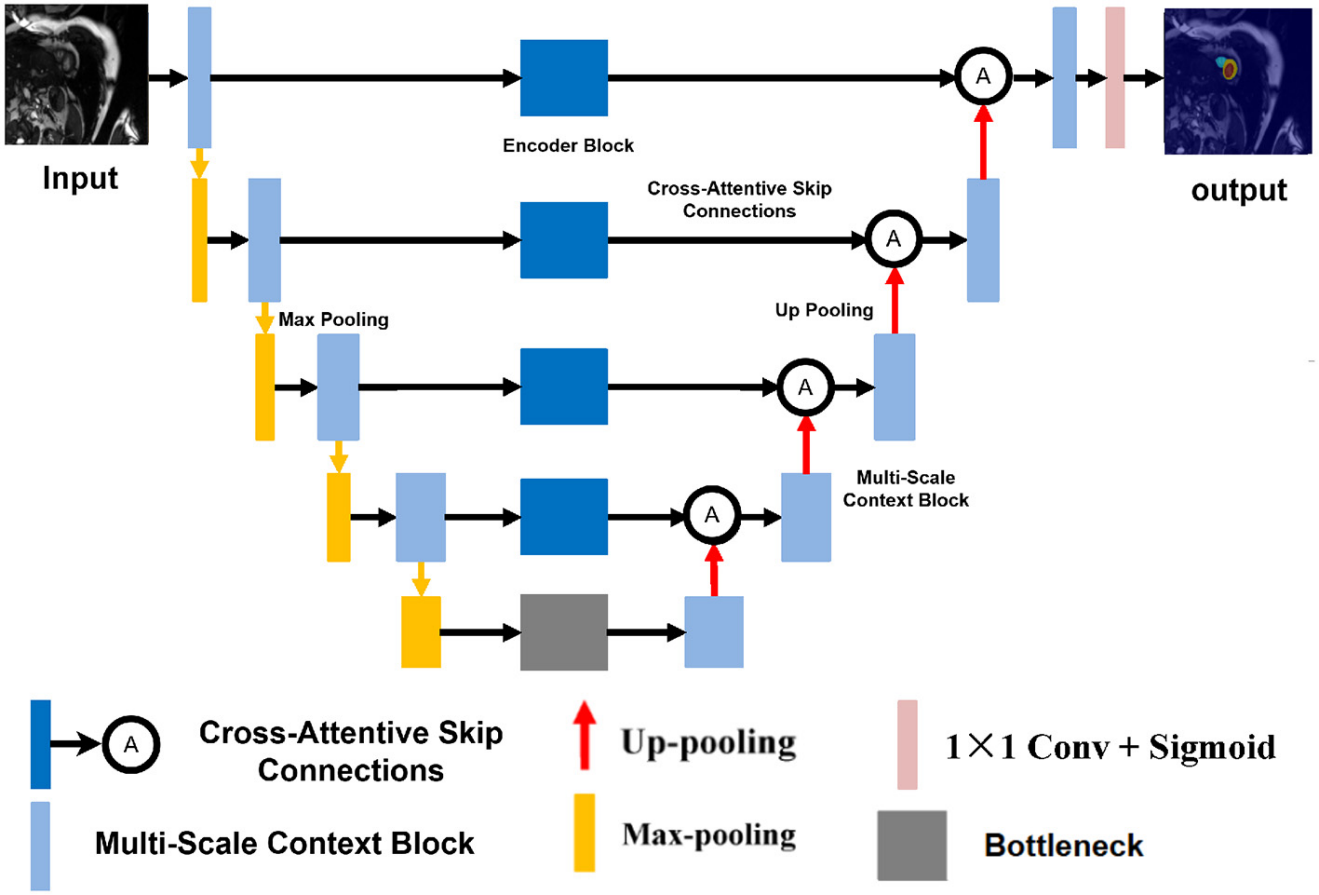
Overview of the proposed CASNet architecture. It consists of a U-Net backbone enhanced with a Multi-Scale Context Block (yellow), Cross-Attentive Skip Connections (orange), and optimized using a Curvature-Aware Loss.

Our proposed architecture, named **CASNet**, is based on the U-Net framework and integrates three key innovations: a Multi-Scale Context Block (MSCB), Cross-Attentive Skip Connections (CASC), and a Curvature-Aware Loss. The overall structure of the network is illustrated in Figure 1.

As shown in Figure 1, the backbone of CASNet follows a standard encoder–decoder topology. The encoder path captures hierarchical semantic features through repeated downsampling and convolutional layers. In the bottleneck region, we incorporate a Multi-Scale Context Block (MSCB) that processes the deepest encoder features using multiple convolution branches with different kernel sizes (e.g., 1 × 1, 3 × 3, and 5 × 5) to enhance receptive field diversity and contextual understanding.

To bridge the semantic gap between encoder and decoder features, we replace the standard skip connections with Cross-Attentive Skip Connections (CASC). Instead of naïvely concatenating feature maps, CASC leverages cross-attention to allow the decoder to selectively query relevant encoder features. This mitigates the mismatch between low-level spatial details and high-level semantic cues. Finally, the entire network is supervised using a composite loss that includes a Curvature-Aware Loss term. This term encourages the predicted contours to be structurally consistent and smooth, which is especially beneficial for cardiac MR segmentation where boundary accuracy is critical.

### 3.2 Multi-scale context block

Convolutional neural networks typically rely on fixed-size kernels, which limits their ability to capture object structures at different spatial scales. This limitation is particularly problematic in medical image segmentation tasks, such as cardiac MRI, where anatomical structures (e.g., the left ventricle, right ventricle, and myocardium) can exhibit significant variation in shape and size across different axial slices. To address this issue, we propose the Multi-Scale Context Block (MSCB), which aims to enrich the semantic representation of the encoder output by aggregating contextual information from multiple receptive fields.

Let ${\text{F}}_{\text{in}}\in {\mathbb{R}}^{C\times H\times W}$ denote the feature map produced by the deepest encoder layer. We process **F**_in_ through multiple parallel convolutional branches with different kernel sizes to capture multi-scale features. Specifically, we define three branches using 1 × 1, 3 × 3, and 5 × 5 convolutions:


$$\begin{array}{r} {\text{F}}_{1}=\sigma \left({\text{Conv}}_{1\times 1}\left({\text{F}}_{\text{in}}\right)\right),\;{\text{F}}_{3}=\sigma \left({\text{Conv}}_{3\times 3}\left({\text{F}}_{\text{in}}\right)\right),\\ {\text{F}}_{5}=\sigma \left({\text{Conv}}_{5\times 5}\left({\text{F}}_{\text{in}}\right)\right) \end{array}$$ (1)

where σ(·) denotes a non-linear activation function, such as ReLU. Each branch is padded appropriately to maintain the spatial size of the input feature map. The outputs of the three branches are concatenated along the channel dimension:


$$\begin{array}{l} {\text{F}}_{\text{cat}}=\text{Concat}\left({\text{F}}_{1},{\text{F}}_{3},{\text{F}}_{5}\right)\in {\mathbb{R}}^{3C\times H\times W} \end{array}$$ (2)

To fuse the multi-scale information and reduce the channel dimension back to *C*, a 1 × 1 convolution is applied:


$$\begin{array}{l} {\text{F}}_{\text{out}}={\text{Conv}}_{1\times 1}\left({\text{F}}_{\text{cat}}\right)\in {\mathbb{R}}^{C\times H\times W} \end{array}$$ (3)

The resulting feature map **F**_out_ contains a rich mixture of fine and coarse contextual cues, enabling the decoder to better resolve ambiguities in anatomical boundaries. This is particularly important in basal and apical slices, where structure shapes are less prominent and harder to distinguish with single-scale representations.

The MSCB is placed at the bottleneck of the network, right after the final encoder layer and before the decoder begins. This ensures that multi-scale contextual information is fully propagated to the upsampling path, enhancing semantic decoding while incurring minimal computational overhead due to the small spatial resolution at this depth. From a functional perspective, the MSCB can be interpreted as a selective enhancement operator:


$$\begin{array}{l} {\text{F}}_{\text{out}}=M\left({\text{F}}_{\text{in}}\right)=\phi \left(\underset{k\in \left\{1,3,5\right\}}{⊕}\sigma \left({\text{Conv}}_{k\times k}\left({\text{F}}_{\text{in}}\right)\right)\right) \end{array}$$ (4)

where ⊕ denotes channel-wise concatenation and ϕ(·) represents the final 1 × 1 convolution used for fusion. This formulation allows the MSCB to act as a multi-scale feature extractor with minimal parameter and memory overhead, while significantly improving the network's ability to handle scale variation and ambiguous regions.

### 3.3 Cross-attentive skip connections

In the standard U-Net architecture, skip connections directly concatenate encoder feature maps with decoder feature maps at corresponding resolution levels. While effective at preserving low-level spatial details, this design assumes that encoder and decoder features are semantically compatible. In practice, this assumption often fails—early encoder layers primarily capture low-level gradients, edges, and textures, while decoder layers operate in a high-level semantic space, refining task-specific predictions. Consequently, naive concatenation may lead to feature misalignment, semantic noise injection, and ultimately degrade segmentation performance.

**Figure 2:**
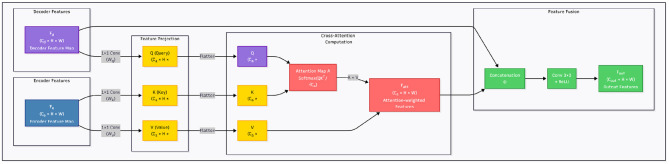
Overview of the Cross-Attentive Skip Connection (CASC) mechanism. Unlike standard skip connections that directly concatenate encoder and decoder features, CASC employs decoder features as queries to extract context-aware features from encoder maps using attention.

To address these limitations, we propose Cross-Attentive Skip Connections (CASC) illustrated in Figure 2, a more flexible and adaptive mechanism that replaces direct concatenation with a cross-attention interaction. The intuition is to allow the decoder to act as a semantic query source, dynamically selecting informative spatial regions from the encoder based on their relevance to the current decoding stage. This enables the network to learn what to skip and what to suppress, promoting more coherent feature fusion.

Formally, let ${\text{F}}_{e}\in {\mathbb{R}}^{C_{e}\times H\times W}$ denote the encoder feature map and ${\text{F}}_{d}\in {\mathbb{R}}^{C_{d}\times H\times W}$ denote the decoder feature map at the same spatial resolution. We first project them into a shared embedding space using learnable 1 × 1 convolutions:


$$\begin{array}{l} \text{Q}=W_{q}*{\text{F}}_{d},\;\text{K}=W_{k}*{\text{F}}_{e},\;\text{V}=W_{v}*{\text{F}}_{e} \end{array}$$ (5)

Here, $\text{Q},\text{K},\text{V}\in {\mathbb{R}}^{C_{a}\times H\times W}$ denote the query, key, and value tensors, respectively. The attention map is computed via scaled dot-product similarity across spatial positions:


$$\begin{array}{l} \text{A}\left(i,j\right)=\frac{\exp\left(\langle {\text{Q}}_{i},{\text{K}}_{j}\rangle /\sqrt{C_{a}}\right)}{\sum_{j^{\prime }}\exp\left(\langle {\text{Q}}_{i},{\text{K}}_{j^{\prime }}\rangle /\sqrt{C_{a}}\right)} \end{array}$$ (6)

The attended feature for each location *i* is given by:


$$\begin{array}{l} {\text{F}}_{\text{att}}\left(i\right)=\underset{j}{\sum }\text{A}\left(i,j\right)\cdot {\text{V}}_{j} \end{array}$$ (7)

This process can be interpreted as the decoder asking: “Which spatial encoder features should I incorporate to reconstruct this region?” Unlike naive skip fusion, the attention map **A** is data-dependent and spatially adaptive, enabling precise alignment between semantic concepts and fine-grained textures.

We then fuse the attention-enhanced encoder features with the decoder features via channel-wise concatenation, followed by a 3 × 3 convolution for feature re-calibration:


$$\begin{array}{l} {\text{F}}_{\text{out}}={\text{Conv}}_{3\times 3}\left(\text{Concat}\left({\text{F}}_{d},{\text{F}}_{\text{att}}\right)\right) \end{array}$$ (8)

This architecture is applied at each resolution level where skip connections are traditionally used. By doing so, the decoder benefits from scale-specific guidance from the encoder while avoiding the semantic conflicts of direct fusion.

From a training perspective, CASC introduces additional non-linearity and information selection pathways that improve gradient flow and regularization. During backpropagation, gradients are weighted by attention relevance, which implicitly suppresses noise in irrelevant regions and encourages the network to focus on informative features. This leads to improved generalization, especially in medical imaging tasks where class imbalance, low contrast, and structural ambiguity are common. CASC transforms skip connections from a rigid structural shortcut into a learnable semantic alignment mechanism. It facilitates fine-to-coarse and coarse-to-fine interactions in a principled manner, improving both the expressiveness and robustness of the segmentation network.

### 3.4 Curvature-aware loss

Standard loss functions for medical image segmentation, such as Dice loss and binary cross-entropy (BCE), primarily focus on per-pixel accuracy and region-level overlap. While effective at aligning global shape, these losses are typically agnostic to the structural continuity and geometric plausibility of predicted contours. In cardiac MRI, where anatomical boundaries like the myocardial wall or endocardium are expected to form smooth, closed curves, traditional pixel-wise supervision often results in jagged or fragmented predictions.

To address this limitation, we incorporate a **Curvature-Aware Loss** that explicitly penalizes local curvature inconsistencies in the predicted segmentation mask. The key idea is to regularize the second-order spatial derivatives of the predicted probability map, encouraging smoothness and continuity along predicted boundaries.

Let $\hat{\text{Y}}\in {\left[0,1\right]}^{H\times W}$ denote the predicted probability map, and **Y**∈{0, 1}^*H*×*W*^ the corresponding ground truth mask. The overall loss function is defined as a weighted combination of three components:


$$\begin{array}{l} L_{\text{total}}=\lambda_{1}\cdot L_{\text{BCE}}+\lambda_{2}\cdot L_{\text{Dice}}+\lambda_{3}\cdot L_{\text{curv}} \end{array}$$ (9)

where λ_1_, λ_2_, λ_3_ are balancing coefficients, and $L_{\text{curv}}$ denotes the curvature-aware term.

We define the curvature-aware loss as the squared Frobenius norm of the image Hessian, capturing local changes in curvature:


$$\begin{array}{l} L_{\text{curv}}=\frac{1}{HW}\underset{i,j}{\sum }||\nabla^{2}\hat{\text{Y}}\left(i,j\right){||}_{F}^{2} \end{array}$$ (10)

Here, $\nabla^{2}\hat{\text{Y}}\left(i,j\right)$ denotes the 2 × 2 Hessian matrix of the prediction at pixel (*i, j*), computed via second-order partial derivatives:


$$\begin{array}{l} \nabla^{2}\hat{\text{Y}}=\left[\begin{array}{cc} \frac{\partial^{2}\hat{\text{Y}}}{\partial x^{2}} & \frac{\partial^{2}\hat{\text{Y}}}{\partial x\partial y}\\ \frac{\partial^{2}\hat{\text{Y}}}{\partial y\partial x} & \frac{\partial^{2}\hat{\text{Y}}}{\partial y^{2}} \end{array}\right] \end{array}$$ (11)

This formulation penalizes high-frequency changes along the predicted boundary and promotes smoother transitions. Operationally, the second-order derivatives can be approximated using discrete Laplacian or finite difference kernels, making the implementation efficient and fully differentiable.

Intuitively, $L_{\text{curv}}$ acts as a geometric regularizer, enforcing that the predicted boundary forms a smooth, natural-looking curve. When combined with region-based losses like Dice and BCE, this loss function ensures both accurate segmentation and anatomically plausible contour shapes.

Empirically, we observe that curvature-aware regularization significantly improves boundary quality, especially in challenging slices with weak edges or low contrast. In addition, this loss term improves robustness to noise and enhances generalization across patients with varying cardiac morphologies.

## 4 Experimental analysis

### 4.1 Datasets

Two publicly available cardiac MRI datasets were utilized to train and evaluate our method. The first dataset is the ACDC-17 dataset, provided by the MICCAI 2017 Automated Cardiac Diagnosis Challenge. It consists of short-axis cardiac cine MRI images acquired from patients with five cardiac conditions: dilated cardiomyopathy (DCM), hypertrophic cardiomyopathy (HCM), myocardial infarction (MI), abnormal right ventricle (ARV), and healthy controls (N). Each case includes end-diastolic (ED) and end-systolic (ES) frames with corresponding manual annotations for the left ventricle (LV), right ventricle (RV), and myocardium (MYO).

The second dataset is M&Ms-2 (Multi-Disease, Multi-View, and Multi-Center), a large-scale cardiac MRI dataset collected from multiple clinical centers using scanners from different vendors. It contains both short-axis (SA) and long-axis (LA) cine sequences, offering higher diversity in imaging protocols and patient pathologies. Similar to ACDC, it provides annotated masks for LV, RV, and MYO in both ED and ES phases.

An overview of the datasets is presented in Table 1.

**Table 1:** Summary of the datasets used in this study.

**Dataset**	**Split (train/test)**	**Categories**	**Labels**	**Images**	**Resolution**
ACDC-17	100/50	DCM, HCM, MI, ARV, N	LV, MYO, RV	1,902	154 × 224 to 428 × 512
M&Ms-2	250/125	DCM, HCM, MI, ARV, N	LV, MYO, RV	1,440 (720 SA)	196 × 240 to 512 × 512

### 4.2 Experimental setup

All experiments were implemented using PyTorch 1.12 and conducted on an NVIDIA RTX 3090 GPU with 24 GB of memory. The training process used the Adam optimizer with an initial learning rate of 1 × 10^−4^, (β_1_, β_2_) = (0.9, 0.999). The learning rate was halved if the validation loss plateaued for more than 10 epochs. Models were trained for 150 epochs with a batch size of 8. Input images were resized to 256 × 256 and normalized to the range [0, 1]. To improve generalization, we applied standard data augmentation techniques during training, including random horizontal and vertical flipping, 0°–15° rotations, scaling within [0.9, 1.1], and elastic deformations. All models, including CASNet and the baseline variants, were trained from scratch under identical conditions. The checkpoint with the best validation performance was used for final evaluation. We employed five-fold cross-validation on both ACDC-17 and M&Ms-2 datasets to ensure robustness. For each fold, 80% of the data was used for training and 20% for testing. All results were averaged across folds. For CASNet, the total loss function combined three components: binary cross-entropy (BCE), Dice loss, and the proposed curvature-aware loss. The weighting coefficients were set to 0.5 for BCE, 0.4 for Dice, and 0.1 for the curvature-aware loss.

### 4.3 Evaluation metrics

To evaluate segmentation performance comprehensively, we adopt several standard metrics: Dice Similarity Coefficient (DSC), Recall, Accuracy (ACC), Precision, Jaccard Index (JC), Hausdorff Distance (HD), and Mean Absolute Distance (MAD). These metrics collectively reflect both region-based overlap and boundary alignment quality.

The Dice Similarity Coefficient (DSC) measures the overlap between the predicted segmentation Ŷ and the ground truth *Y*, and is defined as:


$$\begin{array}{c} \text{DSC}=\frac{2|Ŷ\cap Y|}{\left|Ŷ|+|Y\right|}=\frac{2TP}{2TP+FP+FN} \end{array}$$ (12)

where *TP*, *FP*, and *FN* represent the number of true positives, false positives, and false negatives, respectively.

Recall (sensitivity) and Precision evaluate the completeness and exactness of the segmentation:


$$\begin{array}{c} \text{Recall}=\frac{TP}{TP+FN},\;\text{Precision}=\frac{TP}{TP+FP} \end{array}$$ (13)

Accuracy (ACC) computes the proportion of correctly classified pixels over the entire image:


$$\begin{array}{c} \text{ACC}=\frac{TP+TN}{TP+TN+FP+FN} \end{array}$$ (14)

The Jaccard Index (JC) or Intersection over Union (IoU), quantifies the ratio of the intersection over the union of prediction and ground truth:


$$\begin{array}{c} \text{JC}=\frac{\left|Ŷ\cap Y\right|}{\left|Ŷ\cup Y\right|}=\frac{TP}{TP+FP+FN} \end{array}$$ (15)

Hausdorff Distance (HD) measures the maximum surface distance between the boundary points of the predicted and ground truth masks. Formally, it is defined as:


$$\begin{array}{c} \text{HD}\left(A,B\right)=\max\left\{\underset{a\in A}{\sup}\underset{b\in B}{\inf}||a-b||,\underset{b\in B}{\sup}\underset{a\in A}{\inf}||b-a||\right\} \end{array}$$ (16)

where *A* and *B* are sets of contour points from the predicted and ground truth masks.

Mean Absolute Distance (MAD) captures the average distance between corresponding points on the predicted and ground truth boundaries, given by:


$$\begin{array}{c} \text{MAD}\left(A,B\right)=\frac{1}{\left|A\right|}\underset{a\in A}{\sum }\underset{b\in B}{\min}||a-b|| \end{array}$$ (17)

This metric is more robust to outliers than HD and provides a reliable estimate of average boundary deviation.

### 4.4 Compared models

To validate the effectiveness of the proposed CASNet, we conducted a comprehensive comparative analysis against both traditional CNN-based architectures and recent Transformer-based models for medical image segmentation. All baseline models were trained under identical settings, including data splits, augmentation strategies, loss functions, and training schedules, to ensure a fair and rigorous evaluation.

The following models were selected as benchmarks:

- **U-Net** (23): A classical encoder–decoder architecture with skip connections, which serves as a foundational baseline in medical image segmentation. It is widely adopted due to its simplicity and effectiveness in capturing both low-level spatial details and high-level semantic features.
- **U-Net++** (26): An enhanced version of U-Net that introduces nested and dense skip pathways between encoder and decoder layers, aiming to reduce the semantic gap and improve gradient flow through deep supervision.
- **DeepLabV3** (27): A model that employs atrous spatial pyramid pooling (ASPP) to capture multi-scale contextual information. Although originally designed for natural image segmentation, it has been successfully adapted to various medical imaging tasks.
- **CE-Net** (28): A context encoder network that augments U-Net with a context extraction module based on dilated convolutions and a residual encoder–decoder framework. It is particularly effective at balancing spatial detail preservation and semantic representation.
- **Swin-Unet** (36): A pure Transformer-based architecture that employs shifted window multi-head self-attention for hierarchical representation learning. It represents the state-of-the-art in Transformer-based medical image segmentation.
- **TransUNet** (45): A hybrid CNN-Transformer model that integrates Transformer encoders with U-Net decoders to capture both local texture patterns and global semantic dependencies, demonstrating strong performance on various medical segmentation benchmarks.

All models were re-implemented or adapted using the PyTorch 1.12 framework and trained on the same hardware configuration (NVIDIA RTX 3090 GPU). This controlled experimental setup ensures that any performance differences can be attributed to architectural innovations rather than implementation details or training inconsistencies.

### 4.5 Quantitative results

In this section, we present a comprehensive quantitative evaluation of the proposed CASNet model on two benchmark cardiac MRI datasets: ACDC and M&Ms-2. The results are compared against six baseline methods spanning both traditional CNN architectures (U-Net, U-Net++, DeepLabV3, and CE-Net) and recent Transformer-based models (Swin-Unet, TransUNet). All models were trained under identical experimental conditions to ensure fair comparison.

Performance is reported using seven widely adopted metrics: Dice Similarity Coefficient (DSC), Recall (sensitivity), Accuracy (ACC), Precision, Jaccard Coefficient (JC), Hausdorff Distance (HD), and Mean Absolute Distance (MAD). To assess statistical significance, we conducted paired t-tests comparing CASNet with each baseline method for each metric, with significance levels indicated as: ^*^ (*p* < 0.05), ^**^ (*p* < 0.01), and ^***^ (*p* < 0.001). For distance-based metrics (HD and MAD), lower values are better, so significance indicates CASNet achieved significantly lower (better) values.

#### 4.5.1 Results on ACDC dataset

Table 2 presents the quantitative results on the ACDC dataset. Our proposed CASNet achieves the highest DSC of 96.3%, surpassing TransUNet (95.9%) by 0.4 percentage points, CE-Net (95.6%) by 0.7 percentage points, and substantially outperforming earlier architectures such as Swin-Unet (94.8%), DeepLabV3 (93.5%), U-Net++ (86.8%), and U-Net (85.1%).

**Table 2:** Quantitative comparison on the ACDC dataset.

**Method**	**DSC (%)**	**Recall (%)**	**ACC (%)**	**Precision (%)**	**JC (%)**	**HD (mm)**	**MAD (mm)**
U-Net	85.1 ± 0.9 ^***^	96.6 ± 0.7^**^	76.5 ± 1.8 ^***^	78.2 ± 1.6 ^***^	75.3 ± 0.8 ^***^	13.8 ± 0.8 ^***^	8.6 ± 0.7 ^***^
U-Net++	86.8 ± 0.6 ^***^	96.7 ± 0.8^*^	80.7 ± 0.7 ^***^	82.4 ± 0.8 ^***^	77.1 ± 0.4 ^***^	9.85 ± 0.5 ^***^	6.51 ± 0.6 ^***^
DeepLabV3	93.5 ± 0.6 ^***^	97.2 ± 0.7	88.7 ± 1.1 ^***^	91.0 ± 1.3 ^***^	88.5 ± 0.6 ^***^	2.7 ± 0.7^**^	2.3 ± 0.5 ^***^
Swin-Unet	94.8 ± 0.5^**^	97.4 ± 0.6	96.2 ± 0.7^**^	96.1 ± 0.6^**^	90.3 ± 0.5 ^***^	2.2 ± 0.5^**^	1.9 ± 0.4^*^
CE-Net	95.6 ± 0.3^*^	97.7 ± 0.6	97.6 ± 0.3^*^	97.3 ± 0.5	92.6 ± 0.6	1.9 ± 0.5^*^	1.8 ± 0.4^*^
TransUNet	95.9 ± 0.4^*^	**97.8 ± 0.5**	97.9 ± 0.5	97.1 ± 0.5	92.1 ± 0.4	1.8 ± 0.4	1.6 ± 0.3
**CASNet**	**96.3 ± 0.6**	97.5 ± 0.7	**98.3 ± 1.0**	**97.2 ± 0.6**	**92.4 ± 0.6**	**1.5 ± 0.6**	**1.4 ± 0.5**

Best results in bold. Significance vs. CASNet: ^*^p < 0.05, ^**^p < 0.01, ^***^p < 0.001.

Statistical analysis confirms that CASNet achieves significant improvements across nearly all metrics and baseline comparisons. Notably, while TransUNet demonstrates competitive performance with the highest recall (97.8%), benefiting from its hybrid CNN-Transformer design, CASNet achieves the best overall performance with superior accuracy (98.3%) and significantly better boundary delineation (HD: 1.5 mm, MAD: 1.4 mm). The comprehensive statistical significance across multiple evaluation dimensions validates the robustness of our methodological contributions.

#### 4.5.2 Results on M&Ms-2 dataset

Table 3 summarizes the segmentation results on the M&Ms-2 dataset, which poses additional challenges due to its multi-center, multi-vendor acquisition protocols and greater anatomical diversity. Despite these challenges, CASNet maintains robust generalization performance, achieving a DSC of 95.7%—the highest among all compared methods, with statistically significant improvements across all metrics for most baseline comparisons.

**Table 3:** Quantitative comparison on the M&Ms-2 dataset.

**Method**	**DSC (%)**	**Recall (%)**	**ACC (%)**	**Precision (%)**	**JC (%)**	**HD (mm)**	**MAD (mm)**
U-Net	83.9 ± 1.1^***^	95.8 ± 0.9^***^	75.2 ± 1.5^***^	76.4 ± 1.3^***^	73.0 ± 1.0^***^	14.5 ± 1.0^***^	9.2 ± 0.8^***^
U-Net++	85.5 ± 1.0^***^	95.9 ± 0.8^***^	78.6 ± 1.2^***^	80.5 ± 1.0^***^	74.9 ± 0.9^***^	10.7 ± 0.7^***^	7.1 ± 0.7^***^
DeepLabV3	91.2 ± 0.9^***^	96.4 ± 0.9^**^	86.3 ± 1.3^***^	89.1 ± 1.1^***^	86.2 ± 0.8^***^	3.5 ± 0.8^***^	2.8 ± 0.6^***^
Swin-Unet	89.5 ± 0.8^***^	96.6 ± 0.7^*^	92.1 ± 0.9^***^	93.2 ± 0.8^***^	83.7 ± 0.9^***^	3.8 ± 0.7^***^	3.1 ± 0.6^***^
TransUNet	91.6 ± 0.7^***^	97.2 ± 0.6	93.8 ± 0.8^***^	93.5 ± 0.7^***^	86.8 ± 0.8^***^	3.1 ± 0.6^***^	2.6 ± 0.5^***^
CE-Net	94.1 ± 0.5^**^	97.1 ± 0.7	96.4 ± 0.6^**^	96.6 ± 0.6	90.8 ± 0.7^**^	2.4 ± 0.6	2.1 ± 0.5^*^
**CASNet**	**95.7 ± 0.8**	**97.3 ± 0.8**	**97.5 ± 0.9**	**96.9 ± 0.7**	**91.9 ± 0.8**	**2.1 ± 0.7**	**1.8 ± 0.6**

Best results in bold. Significance vs. CASNet: *p < 0.05, **p < 0.01, ***p < 0.001.

The performance gap is more pronounced on this challenging dataset. CASNet surpasses CE-Net by 1.6 percentage points in DSC and TransUNet by 4.1 percentage points, with highly significant differences (^**^ or ^***^) observed for nearly all metrics. The consistent statistical significance across both overlap-based metrics (DSC, JC, and Precision) and distance-based metrics (HD, MAD) on this diverse dataset validates the superior generalization capability and clinical applicability of our approach.

### 4.6 Computational cost analysis

Although the proposed CASNet introduces additional modules such as cross-attentive skip connections and a multi-scale context block, it remains computationally efficient. To assess the practical feasibility of our approach, we compare the computational cost of CASNet against several widely used baseline models under the same hardware and input settings.

All models were evaluated on an NVIDIA RTX 3090 GPU using 2D cardiac MR images resized to 256 × 256. Table 4 reports the number of trainable parameters, FLOPs per inference, training time per epoch, and inference latency.

**Table 4:** Comparative analysis of computational cost.

**Model**	**Parameters (M)**	**FLOPs (G)**	**Training time/ epoch (min)**	**Inference latency (ms/image)**
U-Net	7.8	25.1	1.9	16.2
U-Net++	9.1	29.3	2.2	18.5
DeepLabV3	11.3	32.6	2.4	19.7
CE-Net	13.4	35.7	2.8	21.1
CASNet (Ours)	**15.2**	**39.1**	**3.0**	**22.8**

All models were evaluated using identical input resolution and batch size. Bold values indicate the proposed CASNet model.

As shown in Table 4 CASNet exhibits a moderate increase in computational demand compared to CE-Net and DeepLabV3, which is expected due to the attention operations. However, the overhead remains within acceptable limits for offline training and inference workflows. The performance gains in segmentation accuracy and boundary quality justify this additional cost in most practical applications.

### 4.7 Ablation study

To assess the individual contributions of each proposed component in CASNet, we conducted a series of ablation experiments. Specifically, we evaluated the effect of the Multi-Scale Context Block (MSCB), Cross-Attentive Skip Connections (CASC), and Curvature-Aware Loss by selectively enabling or disabling them in different variants of the network.

The baseline is a standard U-Net architecture without any of the proposed modules. We then sequentially introduced MSCB, CASC, and the curvature-aware loss, both individually and in combination, while keeping all other training settings constant. This controlled setup enables a clear assessment of each module's impact on segmentation performance. Statistical significance was assessed using paired t-tests comparing each variant against the baseline, with *n* = 100 test cases for ACDC and *n* = 160 test cases for M&Ms-2. Significance levels are denoted as ^*^: *p* < 0.05, ^**^: *p* < 0.01, ^***^: *p* < 0.001. The results are reported on both the ACDC and M&Ms-2 datasets.

To evaluate the contribution of each proposed component, we conduct an ablation study on the ACDC dataset. The experimental results are summarized in Table 5. Starting from the vanilla U-Net baseline, we incrementally add the Multi-Scale Context Block (MSCB), Cross-Attentive Skip Connections (CASC), and Curvature-Aware Loss to analyze their individual and combined effects.

**Table 5:** Ablation study on the ACDC dataset.

**MSCB**	**CASC**	**Curv. Loss**	**DSC (%)**	**Recall (%)**	**ACC (%)**	**Precision (%)**	**JC (%)**	**HD**	**MAD**
✗	✗	✗	85.1 ± 0.9	96.6 ± 0.7	76.5 ± 1.8	78.2 ± 1.6	75.3 ± 0.8	13.8 ± 0.8	8.6 ± 0.7
✓	✗	✗	91.4 ± 0.7^***^	97.0 ± 0.6	86.8 ± 1.2^***^	88.5 ± 0.9^***^	84.8 ± 0.6^***^	4.9 ± 0.6^***^	3.5 ± 0.5^***^
✗	✓	✗	92.7 ± 0.6^***^	97.2 ± 0.7	89.1 ± 1.0^***^	90.3 ± 0.8^***^	86.3 ± 0.6^***^	3.6 ± 0.5^***^	2.9 ± 0.5^***^
✗	✗	✓	90.9 ± 0.8^***^	96.9 ± 0.7	85.2 ± 1.3^***^	87.4 ± 0.9^***^	83.1 ± 0.7^***^	5.4 ± 0.6^***^	3.8 ± 0.6^***^
✓	✓	✗	94.2 ± 0.7^***^	97.3 ± 0.7	94.1 ± 0.8^***^	95.2 ± 0.7^***^	89.5 ± 0.6^***^	2.6 ± 0.6^***^	2.1 ± 0.4^***^
✓	✗	✓	93.5 ± 0.7^***^	97.1 ± 0.6	92.3 ± 0.9^***^	94.4 ± 0.7^***^	88.3 ± 0.6^***^	3.1 ± 0.6^***^	2.4 ± 0.5^***^
✗	✓	✓	94.0 ± 0.6^***^	97.3 ± 0.7	93.0 ± 0.9^***^	94.7 ± 0.7^***^	88.9 ± 0.6^***^	2.8 ± 0.6^***^	2.3 ± 0.4^***^
✓	✓	✓	96.3 ± 0.6^***^	97.5 ± 0.7^*^	98.3 ± 1.0^***^	97.2 ± 0.6^***^	92.4 ± 0.6^***^	1.5 ± 0.6^***^	1.4 ± 0.5^***^

We evaluate the effect of each module: Multi-Scale Context Block (MSCB), Cross-Attentive Skip Connections (CASC), and Curvature-Aware Loss. The symbol ✗indicates the module is disabled and ✓indicates it is enabled. Statistical significance is assessed using paired *t*-test against the baseline. ^*^: *p* < 0.05, ^**^: *p* < 0.01, ^***^: *p* < 0.001.

Introducing MSCB alone significantly improves the Dice score from 85.1% to 91.4% (*p* < 0.001), indicating its effectiveness in capturing contextual features at different scales. Similarly, CASC independently boosts the Dice score to 92.7% (*p* < 0.001), showcasing the importance of semantically aligned skip connections. The Curvature-Aware Loss also contributes positively, increasing the Dice score to 90.9% (*p* < 0.001) and reducing Hausdorff Distance (HD) and Mean Absolute Distance (MAD), particularly enhancing contour smoothness.

Combinations of any two modules yield further improvements, all statistically significant (*p* < 0.001). Notably, combining MSCB and CASC (without the loss term) achieves a Dice of 94.2%, while adding Curvature Loss to either of the blocks provides similar gains in both accuracy and boundary consistency. Incorporating all three modules leads to the best performance across all metrics, with a Dice score of 96.3%, Accuracy of 98.3%, and HD/MAD reduced to 1.5 and 1.4, respectively (all *p* < 0.001 compared to baseline). This clearly demonstrates the complementary nature of the three components in enhancing both region-wise segmentation and structural fidelity.

From Table 6, we observe that all three modules contribute positively to segmentation performance with statistical significance (*p* < 0.001 for all individual modules). Introducing the MSCB improves DSC by over 5% compared to the baseline, indicating its ability to capture multi-scale contextual features. The CASC also offers significant gains in both DSC and boundary accuracy (HD/MAD), demonstrating the importance of semantically guided feature fusion. The Curvature-Aware Loss alone improves contour regularity, evident in the reduction of HD and MAD.

**Table 6:** Ablation study on the M&Ms-2 dataset.

**MSCB**	**CASC**	**Curv. Loss**	**DSC (%)**	**Recall (%)**	**ACC (%)**	**Precision (%)**	**JC (%)**	**HD**	**MAD**
✗	✗	✗	83.9 ± 1.1	95.8 ± 0.9	75.2 ± 1.5	76.4 ± 1.3	73.0 ± 1.0	14.5 ± 1.0	9.2 ± 0.8
✓	✗	✗	89.4 ± 0.9^***^	96.2 ± 0.9	84.5 ± 1.3^***^	85.2 ± 1.1^***^	80.5 ± 0.8^***^	6.9 ± 0.8^***^	4.5 ± 0.6^***^
✗	✓	✗	90.7 ± 0.8^***^	96.3 ± 0.8	86.8 ± 1.2^***^	87.8 ± 1.0^***^	82.3 ± 0.8^***^	5.4 ± 0.7^***^	3.7 ± 0.6^***^
✗	✗	✓	88.6 ± 1.0^***^	96.1 ± 0.9	82.9 ± 1.4^***^	84.2 ± 1.1^***^	79.1 ± 0.9^***^	7.5 ± 0.9^***^	4.8 ± 0.7^***^
✓	✓	✗	92.8 ± 0.8^***^	96.6 ± 0.9	92.2 ± 1.0^***^	93.0 ± 0.8^***^	86.9 ± 0.7^***^	3.0 ± 0.6^***^	2.5 ± 0.5^***^
✓	✗	✓	91.9 ± 0.9^***^	96.4 ± 0.8	90.3 ± 1.1^***^	92.1 ± 0.9^***^	85.7 ± 0.8^***^	3.5 ± 0.6^***^	2.9 ± 0.5^***^
✗	✓	✓	92.5 ± 0.8^***^	96.7 ± 0.9	91.4 ± 1.1^***^	92.7 ± 0.8^***^	86.3 ± 0.7^***^	3.2 ± 0.6^***^	2.6 ± 0.5^***^
✓	✓	✓	95.7 ± 0.8^***^	97.3 ± 0.8^**^	97.5 ± 0.9^***^	96.9 ± 0.7^***^	91.9 ± 0.8^***^	2.1 ± 0.7^***^	1.8 ± 0.6^***^

The symbol ✗indicates the module is disabled and ✓indicates it is enabled. Statistical significance is assessed using paired *t*-test against the baseline. ^*^: *p* < 0.05, ^**^: *p* < 0.01, ^***^: *p* < 0.001.

Combining MSCB and CASC yields a DSC of 92.8% (*p* < 0.001), while pairwise combinations with the curvature loss achieve similar performance levels (all *p* < 0.001). The full model incorporating all three modules achieves the highest performance with a DSC of 95.7%, Accuracy of 97.5%, and substantial reductions in HD and MAD to 2.1 and 1.8, respectively (all *p* < 0.001 compared to baseline). These results confirm the robustness and generalizability of the proposed components across different datasets with varying imaging characteristics.

### 4.8 Qualitative visualization and analysis

**Figure 3:**
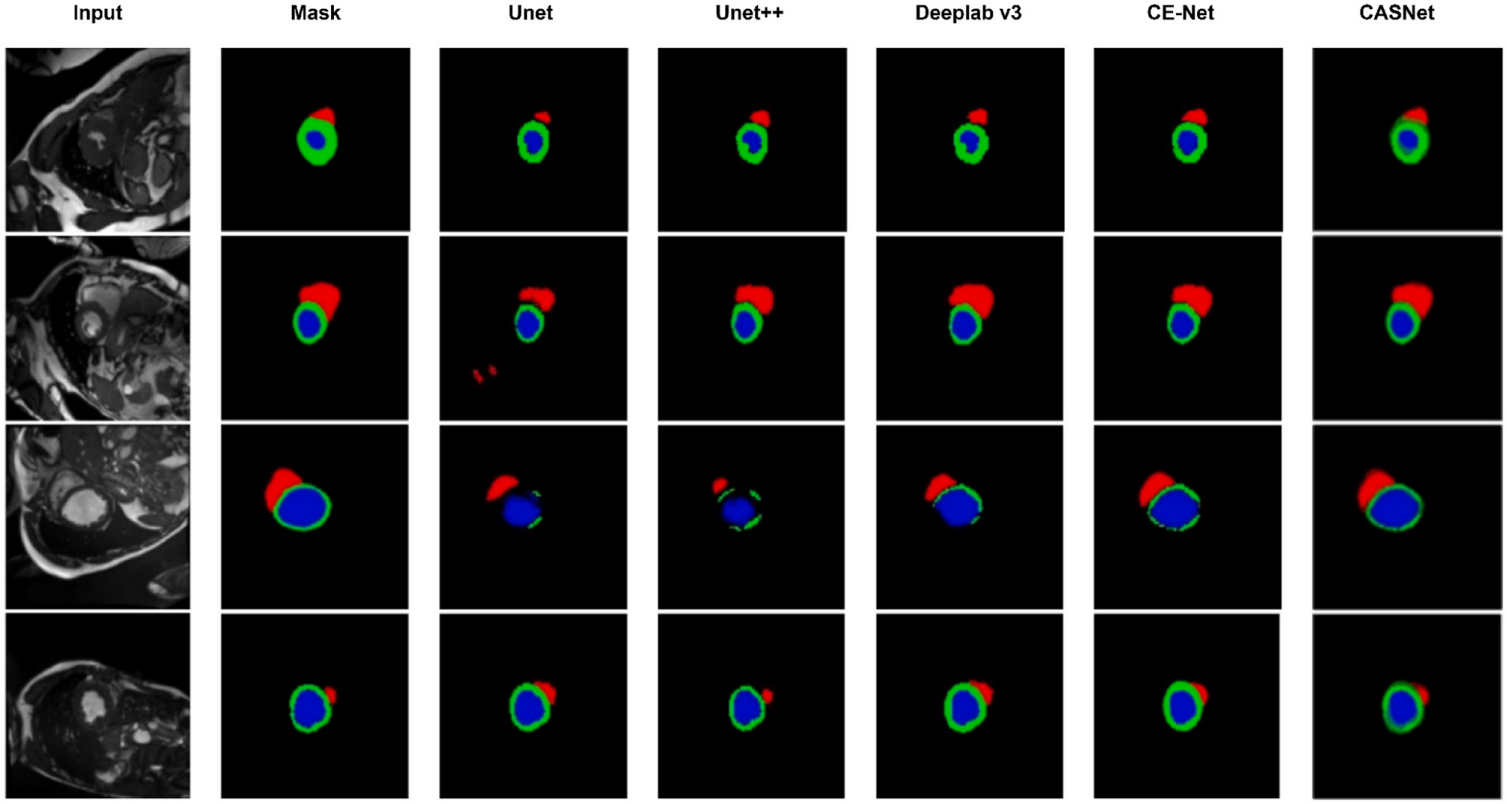
Visual comparison of segmentation results on the ACDC dataset. From **left to right**: Input, Ground Truth, U-Net, U-Net++, DeepLabv3, CE-Net, and our proposed CASNet. CASNet clearly produces more accurate and smooth contours, especially around boundaries and small regions.

**Figure 4:**
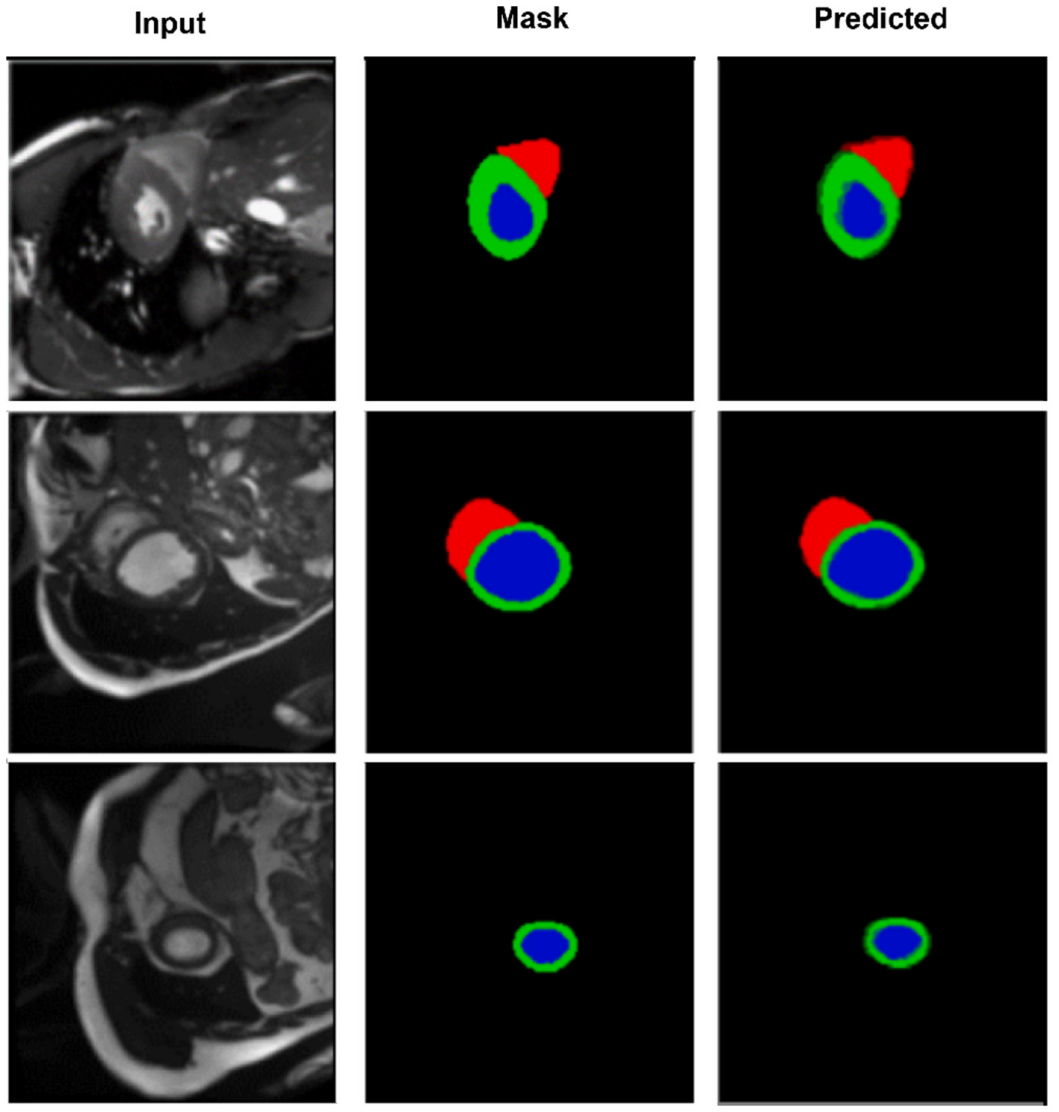
Segmentation results on the M&Ms-2 dataset. Columns represent the input image, ground truth, and output from CASNet. CASNet shows robust generalization and maintains precise delineation across varying patient cases.

To further validate the effectiveness of the proposed method, we present qualitative visual comparisons of segmentation results on both datasets. Figures 3, 4 illustrate sample outputs from multiple baseline models and the proposed CASNet.

As shown in Figure 3, CASNet yields segmentations that are more spatially coherent and closer to the ground truth compared to other methods. For instance, in cases where DeepLabv3 and CE-Net introduce irregular boundaries or miss parts of the myocardium, CASNet successfully preserves anatomical shape with sharper and more consistent predictions. This demonstrates the benefit of the proposed cross-attentive and curvature-aware designs in learning structured features.

Figure 4 further highlights CASNet's robustness under domain variability. Despite differences in contrast and shape, the model consistently segments the left ventricle (blue), myocardium (green), and right ventricle (red) accurately. The smooth contours and structural continuity across frames confirm the model's generalization ability on heterogeneous data.

**Figure 5:**
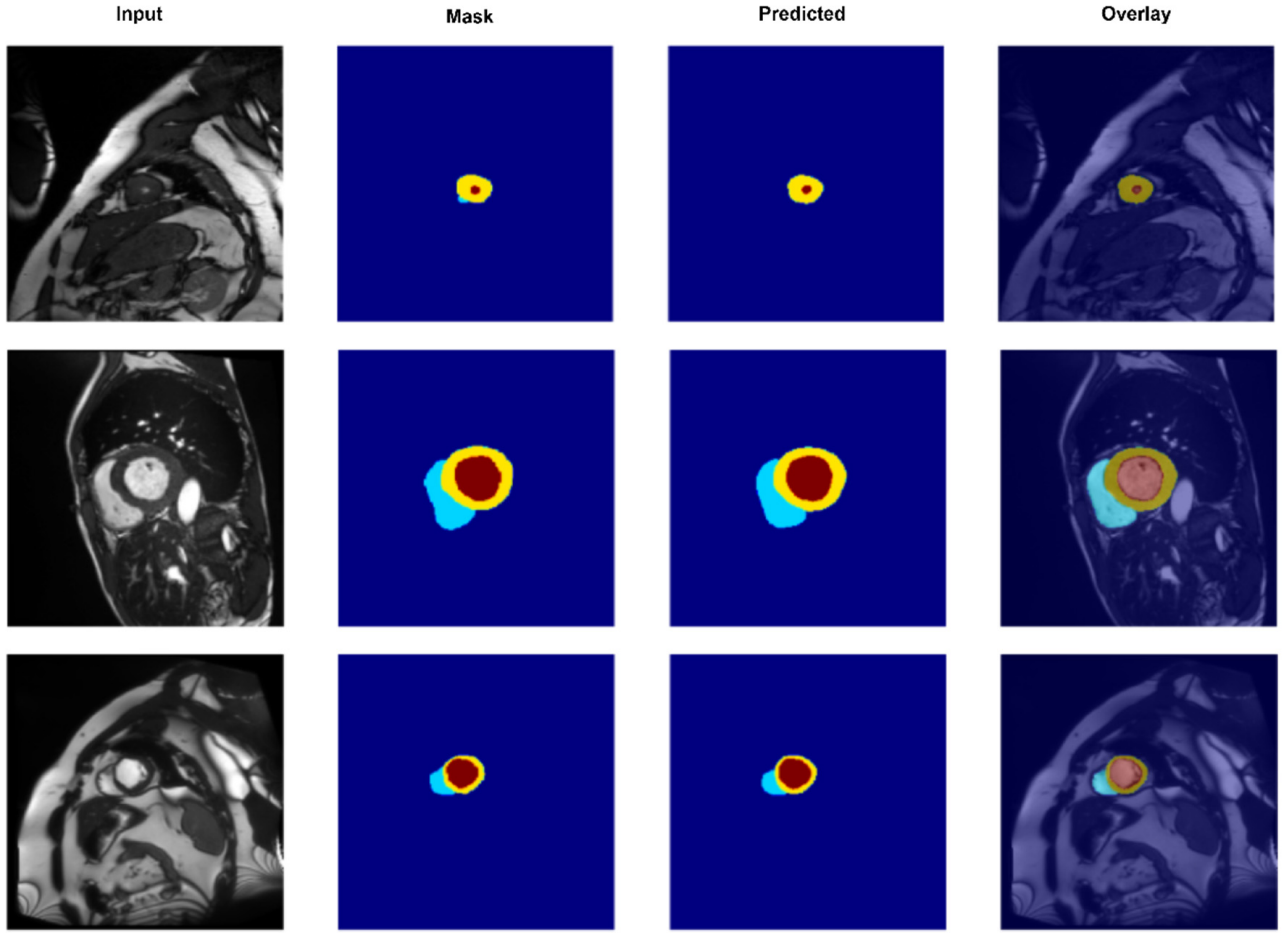
Overlay visualization of segmentation predictions on the ACDC dataset using CASNet. From **left to right**: Input image, ground truth mask, predicted mask, and overlay of prediction on the original input. The color-coded regions represent the right ventricle (cyan), myocardium (yellow), and left ventricle (red).

To gain a deeper understanding of spatial alignment between predicted segmentations and the input anatomy, Figure 5 presents a qualitative overlay of CASNet's predictions on the ACDC dataset. This visualization allows us to evaluate not only the shape fidelity but also the spatial consistency of segment boundaries relative to the original MR images. The overlay columns demonstrate that CASNet is capable of producing highly aligned and anatomically plausible contours. In all three examples, the boundaries between the myocardium, left ventricle, and right ventricle closely follow the true anatomical structures visible in the grayscale input. Notably, regions with tight curvature or close class adjacency are well captured without excessive bleeding or class mixing.

### 4.9 External validation on Sunnybrook dataset

To further assess the generalization capability of CASNet to unseen clinical centers and acquisition protocols, we conducted external validation on the Sunnybrook Cardiac Dataset. This dataset comprises 45 cardiac MRI cases acquired at an independent institution with different imaging parameters and patient demographics. We applied the models trained on the ACDC training set directly to all Sunnybrook subjects without any fine-tuning, domain adaptation, or hyperparameter modification. This zero-shot cross-domain evaluation protocol rigorously assesses the intrinsic robustness of learned representations when transferred to a completely unseen imaging domain.

Table 7 presents an ablation analysis on the Sunnybrook dataset using models trained on ACDC, revealing the contribution of each component to cross-domain generalization. The baseline U-Net trained on ACDC achieves 74.3% DSC when directly applied to Sunnybrook, establishing a lower bound for zero-shot performance. Incorporating standard skip connections improves DSC to 76.8%, demonstrating the benefit of multi-level feature fusion for domain transfer. The model with Cross-Attentive Skip Connections (CASC) further boosts performance to 78.5%, indicating that adaptive feature recalibration enhances robustness to domain variations. Adding the Multi-Scale Context Block (MSCB) yields 80.2% DSC, showing that multi-scale contextual modeling improves generalization across different imaging protocols. The full CASNet with Curvature-Aware Loss (CAL) achieves 82.4% DSC and 70.2% JC, representing an 8.1 percentage point improvement in DSC over the baseline and demonstrating that explicit boundary regularization contributes to cross-domain robustness.

**Table 7:** Ablation study on Sunnybrook dataset using models trained on ACDC (zero-shot cross-domain evaluation).

**Model variant**	**CASC**	**MSCB**	**CAL**	**DSC (%) ↑**	**JC (%) ↑**	**HD (mm) ↓**	**MAD (mm) ↓**
Baseline (U-Net)	✗	✗	✗	74.3 ± 2.8	59.8 ± 3.2	12.5 ± 2.1	7.8 ± 1.9
+ Skip Connections	✗	✗	✗	76.8 ± 2.5	62.7 ± 2.9	11.2 ± 1.9	6.9 ± 1.7
+ CASC	✓	✗	✗	78.5 ± 2.3	65.1 ± 2.7	9.8 ± 1.7	5.7 ± 1.5
+ CASC + MSCB	✓	✓	✗	80.2 ± 2.1	67.3 ± 2.4	8.6 ± 1.5	4.9 ± 1.3
CASNet (Full)	✓	✓	✓	**82.4 ± 1.9**	**70.2 ± 2.2**	**7.4 ± 1.3**	**4.1 ± 1.1**

The symbol ✗indicates the module is disabled and ✓indicates it is enabled. ↑ indicates higher values are better; ↓ indicates lower values are better. Bold values indicate the best performance achieved by the full CASNet model.

Comparing the zero-shot performance on Sunnybrook (82.4% DSC) with the in-distribution performance on ACDC test set (96.3% DSC), CASNet exhibits a 13.9 percentage point degradation, which is expected given the substantial domain shift between the two datasets. Notably, the incremental contributions of CASC, MSCB, and CAL remain relatively consistent across domains, validating that our architectural innovations promote learning of domain-invariant features rather than dataset-specific patterns. The Hausdorff Distance and Mean Absolute Distance metrics show pronounced improvements with each component addition, with the full CASNet achieving 7.4 mm HD and 4.1 mm MAD. The Curvature-Aware Loss yields substantial improvement in boundary metrics even under domain shift, confirming its effectiveness in maintaining edge quality across different imaging conditions. These results demonstrate that CASNet's design choices collectively contribute to robust cross-domain generalization, supporting its potential for clinical deployment across diverse imaging centers without requiring site-specific retraining.

## 5 Conclusion

In this work, we proposed CASNet, a novel deep learning architecture for accurate and anatomically consistent cardiac MRI segmentation. CASNet integrates three key innovations into a U-Net backbone: a Multi-Scale Context Block (MSCB) to enhance contextual representation at the encoder bottleneck, Cross-Attentive Skip Connections (CASC) to bridge semantic gaps between encoder and decoder layers, and a Curvature-Aware Loss to regularize boundary smoothness and structural fidelity. Extensive experiments conducted on two benchmark datasets, ACDC and M&Ms-2, demonstrate that CASNet consistently outperforms established baselines. Ablation studies further confirm the complementary roles of each proposed component, with each module contributing independently and synergistically to the final performance. Qualitative visualizations also highlight CASNet's ability to preserve anatomical integrity and generate smooth, precise segmentation masks even in complex or low-contrast regions.

Despite its strong performance, CASNet has several limitations. First, the cross-attention mechanism introduces additional computational overhead, which may limit its real-time applicability on resource-constrained devices. Second, while our curvature-aware loss improves boundary quality, it relies on accurate second-order derivative approximations, which may be sensitive to image noise or aliasing. Third, CASNet was trained and evaluated on 2D slices, which may limit its ability to fully capture 3D anatomical continuity across frames in cine-MRI sequences. Furthermore, although the model generalized well across two datasets, broader validation on additional centers and imaging protocols is needed to confirm clinical robustness.

Future work will pursue several promising directions to address these limitations and extend the capabilities of CASNet. To leverage volumetric information, we plan to develop a 3D variant of CASNet by replacing 2D convolutions with 3D counterparts in the encoder and decoder, enabling the model to capture inter-slice dependencies and improve spatial coherence across cardiac volumes. For temporal consistency in cine-MRI sequences, we will explore integrating recurrent modules such as ConvLSTM or temporal attention mechanisms into the decoder to model cardiac motion dynamics across consecutive frames. To reduce computational cost for clinical deployment, we will investigate model compression techniques including knowledge distillation, where a compact student network learns from the full CASNet teacher, and neural architecture search to identify efficient architectures that balance accuracy and inference speed. Additionally, we will explore hybrid architectures that combine lightweight convolutional backbones with selective attention modules to maintain performance while reducing parameter count. To improve cross-domain generalization, we plan to incorporate unsupervised domain adaptation methods such as adversarial training or style transfer, enabling the model to adapt to new imaging protocols without requiring labeled data from each site. Finally, integrating anatomical shape priors through learned shape embeddings or atlas-based regularization may further enhance structural consistency and robustness to pathological variations. Preliminary experiments suggest that these directions are both feasible and promising for advancing CASNet toward broader clinical applicability.

## Data Availability

The original contributions presented in the study are included in the article/supplementary material, further inquiries can be directed to the corresponding author.
